# Temporal fluctuations of post-tonsillectomy haemorrhage

**DOI:** 10.1007/s00405-021-07080-1

**Published:** 2021-09-23

**Authors:** Stefan Grasl, Patrick Mekhail, Stefan Janik, Christoph M. Grasl, Erich Vyskocil, Boban M. Erovic, Christoph Arnoldner, Lukas D. Landegger

**Affiliations:** 1 Department of Otorhinolaryngology, Head and Neck Surgery, Medical University of Vienna, Vienna, Austria; 2 Institute of Head and Neck Diseases, Evangelical Hospital, Vienna, Austria

**Keywords:** Tonsillectomy, Post-tonsillectomy haemorrhage, Circadian rhythm, Seasons, Revision surgery

## Abstract

**Purpose:**

Although haemorrhage is a common and in some cases life-threatening complication after tonsillectomy, surprisingly little is known about the temporal fluctuations of the onset of bleeding. The purpose of this study was to assess circadian and seasonal rhythms of post-tonsillectomy haemorrhage (PTH) and potential ramifications to educate patients and health care staff.

**Methods:**

This retrospective study carried out at a tertiary referral hospital included paediatric and adult patients requiring emergency surgery due to severe PTH between 1993 and 2019. Medical records were reviewed and patient demographics, details regarding the initial procedure, postoperative day of haemorrhage, and start time of emergency surgery were extracted. Descriptive statistics, Kruskal–Wallis test, Mann–Whitney *U* test, and Chi-square goodness of fit tests were used to detect potential differences.

**Results:**

A total of 300 patients with severe PTH and subsequent emergency surgery were identified. The median postoperative duration until PTH was 6 (range: < 1–19) days. 64.7% (*n* = 194) of all emergency surgeries had to be performed during evening and night hours (6 pm—6 am) (*p* < 0.0001). Compared to diurnal incidents, the risk of a nocturnal PTH event increased, the longer ago the initial surgery was (*p* < 0.0001). No seasonal variations were identified. Age, sex, and details of the initial procedure had no significant influence on the start time according to the surgical protocol.

**Conclusion:**

The discovered temporal fluctuations of PTH are of relevance for patient awareness and preoperative education. Due to possible life-threatening complications, management of severe PTH requires specific resources and trained medical staff on call.

## Introduction

Tonsillectomy is one of the most frequently performed surgical procedures in otolaryngology. Post-tonsillectomy haemorrhage (PTH) is a serious and potentially lethal complication with an overall reported risk between 2 and 21.4% [1–5]. Among those affected by such an event, up to 5% experience severe PTH with a need for surgical revision under general anaesthesia [1, 4–6]. Primary PTH occurs within the first 24 h postoperatively and is mainly attributed to surgical factors, while secondary PTH typically begins 5–10 days postoperatively [1, 4–8]. Although the aetiology of PTH is not completely understood, increased efforts are being made to better grasp relevant factors for PTH and subsequently reduce morbidity and mortality [8–12].

Temporal fluctuations have been shown to play a role in a wide variety of bleeding incidents, e.g., in the occurrence and pathophysiology of myocardial infarctions, strokes, and venous thromboembolism [13–18].

To our knowledge, the first study focusing on the influence of the circadian rhythm on PTH was published by D’Agostino et al. with the description of 59 bleeding events in children (average age 6.8 years) who had undergone adenotonsillectomy or tonsillectomy [19]. The researchers detected an increased occurrence of PTH between 9 pm and 9 am with two specific peaks, namely from 10 pm to 1 am and from 6 to 9 am.

Another study attempting such an analysis for PTH in several adults and again mostly paediatric patients (mean age 17.1 years) was published in 2019 by Kim et al. After analysing 120 patients with PTH, 104 of them had emergency department arrival times and 60 could provide the exact timing of bleed onset. In the latter group, the researchers detected a circadian variation, namely an increased incidence of secondary bleeding complications in the period between midnight and 6 am [12].

As both of these research groups assessed a relatively limited number of incidents of different severity, the aim of the present study was to verify the validity of their findings in a larger and more homogenous cohort of patients. In particular, we were interested in subjects that require treatment in the operating room and thus necessitate substantial resources and immediate attention. Furthermore, because the other two studies primarily included children, a focus on adult patients was anticipated and as a supplementary goal, seasonal variations of the bleeding episodes should be assessed as well. In general, evaluation of additional data and variables is expected to lead to a better understanding concerning the onset of a PTH event, subsequently facilitating more precise expectations of patients and caregivers with a potential reduction of morbidity and mortality.

## Materials and methods

We performed a retrospective chart review of all patients (*n* = 300) who had undergone emergency surgery exclusively due to PTH between December 1993 and November 2019 at our department.

All initial scheduled surgical interventions (*n* = 8965) had been performed under general anesthesia, using cold-steel dissection and haemostasis with bipolar diathermy. As a result of this tertiary referral hospital’s structure, all planned surgical procedures had been carried out between 8 am and 3 pm. Patients were hospitalized on average for three nights. Seven to ten days after surgery, a routine follow-up examination in the outpatient clinic was performed.

A subset of patients had undergone initial surgery at other institutions and was directly brought to our department’s emergency room due to severe PTH events. Additional subanalyses were carried out for patients with bleeding after tonsillectomy, tonsillectomy à chaud, uvulopalatopharyngoplasty (UPPP), or combined adenotonsillectomy (T&A). Patients with a PTH event after a tonsillectomy for diagnosis/treatment of tonsillar malignancy or without a recorded bleeding time were excluded.

### Data collection

Patients’ records were retrospectively evaluated to retrieve patient age (at time of surgery) and sex, treating institution, date, and type of initial surgery, postoperative day of haemorrhage, as well as the start time of emergency surgery. Onset of haemorrhage (based on the start time of emergency surgery) was primarily categorized into two groups: diurnal (6:01 am–6:00 pm) and nocturnal (6:01 pm–6:00 am), but also further subcategorized into the four groups established by Kim et al. [12]: evening (6:01 pm–12 am), night (12:01 am–6 am), morning (6:01 am–12 pm), and afternoon (12:01 pm–6 pm).

The postoperative period was divided into four phases according to patient discharge schedule and the stages of wound healing [20, 21]: 0–24 h post surgery, 1–3 days, 4–10 days, and the period after the 10^th^ postoperative day.

### Statistical analysis

Statistical analyses were performed using SPSS Version 26.0 software (IBM Corp. Armonk, NY, USA). Graphical illustrations were performed using Microsoft Excel 2016. Data are presented as n (%) or median with IQR (interquartile range) within the results section.

Chi-square goodness of fit test was used to assess the temporal distribution of haemorrhage and Chi-square test was carried out to compare nominal variables. Graphical illustration and Kolmogorov–Smirnov test were performed to analyse normal distribution and Kruskal–Wallis test together with Mann–Whitney-*U* test were used to compare means of two or more independent groups.

All tests were two-sided and *p*-values below 0.05 were considered as statistically significant.

## Results

A total of 300 patients, including 132 (44.0%) females and 168 (56.0%) males, with a median age of 21.0 years (IQR: 17.8 y; range: 3–77 y) were analysed. All had severe PTH requiring emergency surgery and revision. 238 (79.3%) patients had undergone the primary intervention at our own department and 62 (20.7%) at another institution. For 210 (70.0%) patients, the primary surgery had been routine tonsillectomy, for 12 (4.0%) tonsillectomy à chaud, for 4 (1.3%) uvulopalatopharyngoplasty (UPPP), and 74 (24.7%) underwent combined adenotonsillectomy (T&A) (Table 1).

**Table 1:** Patient characteristics by the onset of post-tonsillectomy haemorrhage

	Overall	Morning (6:01 am– 12 pm)	Afternoon (12:01 pm– 6 pm)	Evening (6:01 pm– 12 am)	Night (12:01 am– 6 am)	*p*-value	Diurnal (6:01 am– 6:00 pm)	Nocturnal (6:01 pm– 6:00 am)	*p*-value
	300 (100)	43 (14.3)	63 (21.0)	104 (34.7)	90 (30.0)	< 0.0001^a^	106 (35.3)	194 (64.7)	< 0.0001^a^
*Age*									
Overall (years)	21.0 [IQR: 17.8]	22.0 [IQR: 22.0]	22.0 [IQR: 14.0]	20.0 [IQR: 17.5]	20.5 [IQR: 15.3]	0.443^b^	22 [IQR: 19.3]	20 [IQR: 16.3]	0.167^c^
*Age groups*									
2–5 years	28 (9.3)	5 (17.9)	4 (14.3)	9 (32.1)	10 (35.7)		9 (32.1)	19 (67.9)	
≥ 6–12 years	53 (17.7)	9 (17.0)	9 (17.0)	22 (41.5)	13 (24.5)		18 (34.0)	35 (66.0)	
≥ 13–17 years	46 (15.3)	4 (8.7)	8 (17.4)	18 (39.1)	16 (34.8)		12 (26.1)	34 (73.9)	
≥ 18 years	173 (57.7)	25 (14.5)	42 (24.3)	55 (31.8)	51 (29.5)	0.705 ^d^	67 (38.7)	106 (61.3)	0.430^d^
*Sex*									
Female	132 (44.0)	15 (11.4)	27 (20.5)	53 (40.2)	37 (28.0)		42 (31.8)	90 (68.2)	
Male	168 (56.0)	28 (16.7)	36 (21.4)	51 (30.4)	53 (31.5)	0.280 ^d^	64 (38.1)	104 (61.9)	0.259^d^
*Type of primary intervention*									
Tonsillectomy	210 (70.0)	30 (14.3)	46 (21.9)	69 (32.9)	65 (31.0)		76 (36.2)	134 (63.8)	
Tonsillectomy à chaud	12 (4.0)	2 (16.7)	3 (25.0)	4 (33.3)	3 (25.0)		5 (41.7)	7 (58.3)	
Uvulopalatopharyngoplasty	4 (1.3)	0 (0.0)	1 (25.0)	1 (25.0)	2 (50.0)		1 (25.0)	3 (75.0)	
Adenotonsillectomy (T&A)	74 (24.7)	11 (14.9)	13 (17.6)	30 (40.5)	20 (27.0)	0.955^d^	24 (32.4)	50 (67.6)	0.864^d^
*Location of primary surgery*									
Local department	238 (79.3)	34 (14.3)	54 (22.7)	79 (33.2)	71 (29.8)		88 (37.0)	150 (63.0)	
Other department	62 (20.7)	9 (14.5)	9 (14.5)	25 (40.3)	19 (30.6)	0.513^d^	18 (29.0)	44 (71.0)	0.244^d^
*Season*									
Winter	69 (23.0)	8 (11.6)	19 (27.5)	24 (34.8)	18 (26.1)		27 (39.1)	42 (60.9)	
Spring	90 (30.0)	14 (15.6)	18 (20.0)	30 (33.3)	28 (31.1)		32 (35.6)	58 (64.4)	
Summer	74 (24.7)	6 (8.1)	12 (16.2)	28 (37.8)	28 (37.8)		18 (24.3)	56 (75.7)	
Autumn	67 (22.3)	15 (22.4)	14 (20.9)	22 (32.8)	16 (23.9)	0.289^d^	29 (43.3)	38 (56.7)	0.102^d^

Data are presented as *n* (%) or median [interquartile range]; ^a^Chi-square goodness of fit test, ^b^Kruskal-Wallis test, ^c^Mann-Whitney-*U* test, ^d^Chi-square test

### General temporal fluctuations of PTH

The median postoperative duration until PTH was 6 days (IQR: 5 d; range: < 1–19 d). 66.7% of all PTH events (*n* = 200) occurred between 4th and 10th postoperative day. 24 (8.0%) had a primary PTH within the first 24 h after surgery and 40 patients (13.3%) developed haemorrhage between 24 and 72 h postoperatively. 12% of all PTH events (*n* = 36) occurred more than 10 days after the primary surgery and the latest on the 19^th^ postoperative day. Age, sex, or type of primary intervention had no significant influence on the postoperative duration until PTH (Table 1).

14 (4.7%) patients had two or more severe PTH events and had to undergo a second revision under general anaesthesia. The median duration between the first PTH event and the second emergency surgery was 4 days (IQR: 5 d) and between initial surgery and the second PTH event 10 days (IQR: 8 d).

### Seasonal variation of PTH

We further analysed a potential correlation between the seasons and risk of PTH. Overall, 69 (23.0%), 90 (30.0%), 74 (24.7%), and 67 (22.3%) individuals had undergone emergency surgery in winter, spring, summer, and autumn, respectively. Hence, no significant difference regarding the frequency of PTH and season could be detected (*p* = 0.289). This conclusion did not change when correcting for seasonal variations of the number of tonsillectomies carried out at our own department: relative percentages of PTH, dependent on the season, were ranging between 2.3% and 3.0% (*p* = 0.502).

### Circadian variation of PTH

**Fig. 1:**
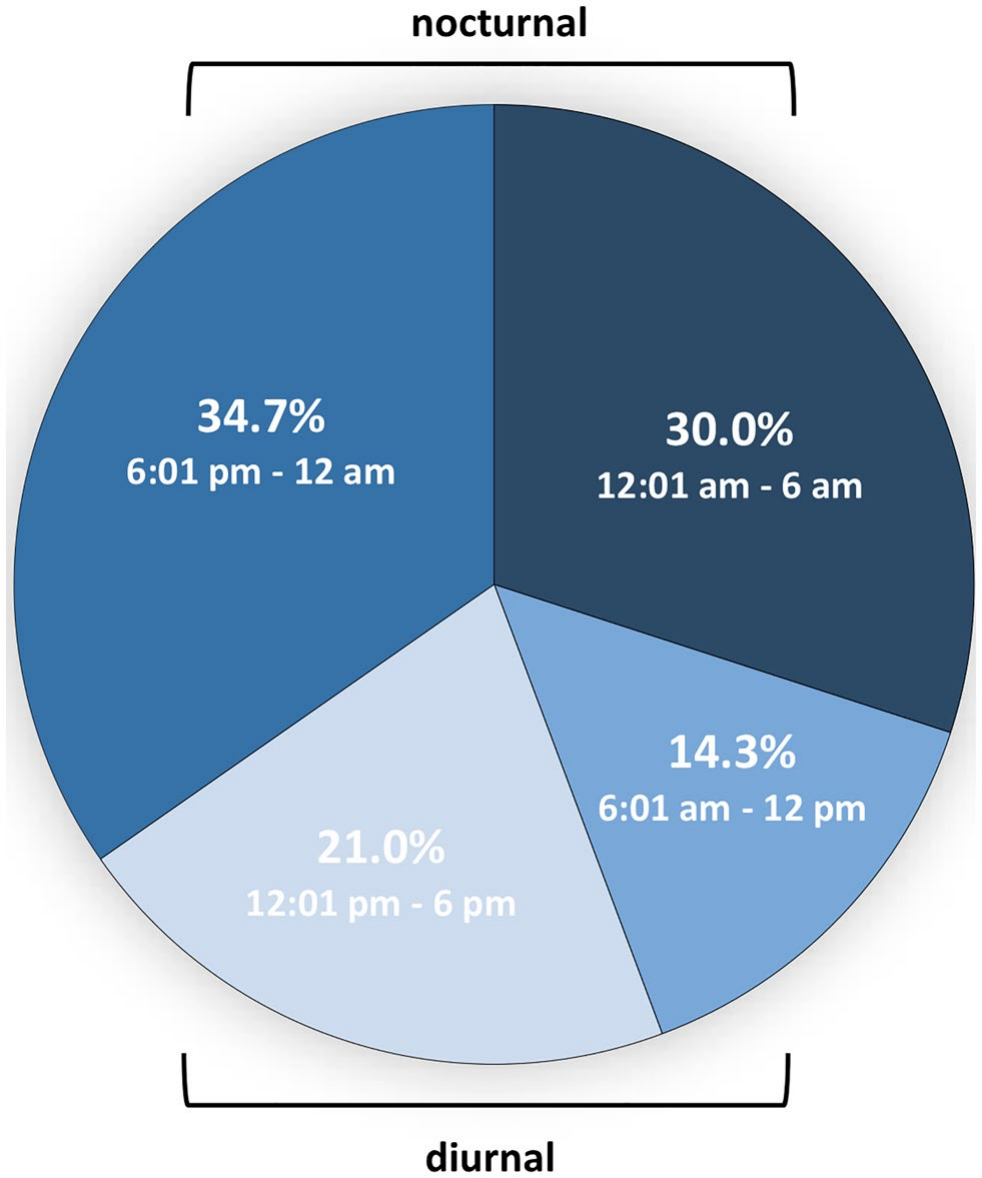
Circadian variation of severe post-tonsillectomy haemorrhage based on start time of emergency surgery

Among the 300 patients with severe PTH, 43 (14.3%) underwent emergency surgery in the morning, 63 (21.0%) in the afternoon, 104 (34.7%) in the evening, and 90 (30.0%) at night (*p* < 0.0001) (Fig. 1).

When using the diurnal/nocturnal dichotomy, overall, 64.7% (*n* = 194) of all emergency surgeries due to PTH had to be performed overnight (6:01 pm–6:00 am).

In addition, 64.3% (*n* = 9) of patients who had two or more severe PTH events had to undergo a second nocturnal revision under general anaesthesia (6:01 pm–6:00 am).

### Diurnal/nocturnal onset of PTH in context of postoperative duration

**Fig. 2:**
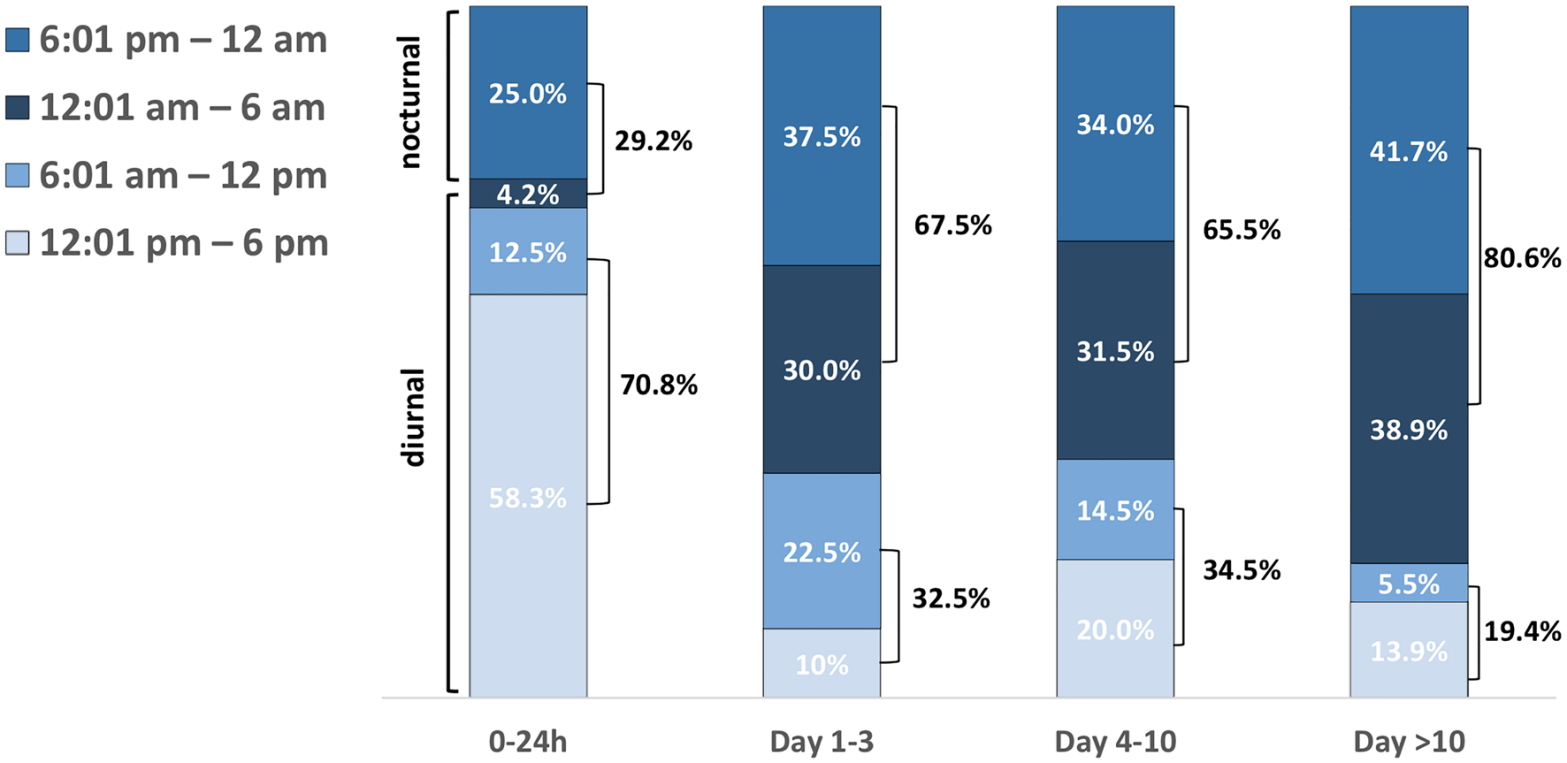
Distribution of diurnal and nocturnal severe post-tonsillectomy haemorrhage events categorized according to onset/emergency surgery start time and in relation to the postoperative period

We further analysed a potential association between the start time of emergency surgery due to PTH and the extent of postoperative duration after the primary surgery. With increasing time after initial surgery, the rate of nocturnal PTH events (6:01 pm–6:00 am) increased from 29.2% during the first 24 h (versus a diurnal incident, 6:01 am–6:00 pm) up to 80.6% after the 10th postoperative day (*p* < 0.0001). For further details see Fig. 2.

## Discussion

Postoperative haemorrhage after tonsillectomy or one of the other routine surgeries named above is common and sometimes fatal [1–6].

According to published literature [7, 8], the highest frequency of PTH is around postoperative days 5-7, which could be confirmed in our patient population. After the scientific community had gained initial insights into the occurrence of PTH, concepts of postoperative management after tonsillectomy were implemented at many centres, yet several unknowns and regional differences remained. E.g., in view of the growing demand for cost-effectiveness in health care systems [22], postoperative length of hospital stay varies significantly between countries and institutions. In particular, these differences range from outpatient tonsillectomy to postoperative inpatient care for several days [22–27]. Independent of the chosen approach, in the present study, we set out to analyse temporal fluctuations of PTH events to streamline various aspects prior to, during, and after tonsillectomy, providing valuable information for physicians, patients, and administrators alike.

Based on the currently available literature, it is still unclear whether there are seasonal differences in the rate of PTH events. While some authors could detect fluctuations [28, 29], the results of this and other studies do not hint at such a correlation [30–32]. Because the studies were all carried out in geographically distinct locations, country-specific seasonal variations might play a role.

Yet, our analyses revealed a significant accumulation of PTH events overnight, which had also been shown by D’Agostino et al. and Kim et al. in exclusively or primarily paediatric patient cohorts [12, 19]. One hypothesis explaining this finding is that circadian changes of blood viscosity and coagulation factors result in prolonged clotting at certain times [33]. Nevertheless, in rats, nocturnal coagulation took significantly longer, which would be the opposite in humans as our species is diurnal [34].

However, significant differences compared to the works of D’Agostino et al. and Kim et al. have to be mentioned [12, 19]. First, we decided to include only patients with a severe PTH event and subsequent emergency surgery. With this selection strategy, we were able to ensure the exact time of haemorrhage, which is (a) more precise than just relying on patient history and (b) more relevant for health care providers. Second, only a homogenous patient population was included, particularly with active haemorrhage (at least grade III on the post-tonsillectomy bleeding scale suggested by Walner et al. [35]) necessitating transfer to the operating theatre and thus requiring and tying up key resources. Although we used the above-mentioned stringent inclusion criteria, our analysed patient collective is at least five times as large as the ones described by Kim et al. and D’Agostino et al. Yet, we do not only substantiate the conclusions of the other two groups regarding the paediatric patient population, but also add valuable information by presenting data focused on adults (173 individuals or 57.7%). While the analysis of PTH events of our 300 patients also showed a highly significant rise in nocturnal frequency of bleeding, we could additionally demonstrate that with longer duration after initial surgery, the rate of PTH events during the night increased even more compared to diurnal episodes—adding pertinent details relevant to the preoperative informed consent and postoperative management. As shown in a recent publication, preoperative tonsillectomy education is important for decreasing adverse postoperative outcomes and associated healthcare costs [36]. Since most severe bleeding episodes are non-preventable complications, a better understanding of temporal fluctuations of PTH should enhance general awareness and improve reaction times of patients and treating physicians to prevent fatal outcomes. Because time is crucial in case of severe bleeding, we are convinced that these findings are not only of utmost importance for otolaryngologists, but also for emergency physicians, paramedics, and other medical staff, particularly of emergency rooms, operating theatres and paediatric and/or surgical wards. Severe PTH events, especially during the night, often require an interdisciplinary management and treatment due to limited resources and operating theatre capacities. Surgical treatment of such complications can be very demanding, sometimes even requiring carotid artery exploration and ligation.

As a result, reducing resources or training for medical staff in times of growing demand for cost-effectiveness can in the worst case result in an increase of life-threatening or fatal PTH events, especially in peripheral non-urban areas with a low density of medical facilities as well as longer ambulance response and patient transfer times.

Despite its retrospective nature and inherent bias of selection, we anticipate that the results of our study add important information to the education and awareness of physicians and patients in general and particularly to emergency PTH management to make tonsillectomy safer in the future.

## Data Availability

The datasets used and/or analysed during the current study are available from the corresponding author on request.
